# 83 Topical Tranexamic Acid on Skin Graft Recipient Sites in Burn Reconstruction

**DOI:** 10.1093/jbcr/iraf019.083

**Published:** 2025-04-01

**Authors:** Matthew DePamphilis, Thomas Guzman, Hilary Kusz, Nikki Rosado, Tina Moon, Derek Hursey, Daniel Driscoll

**Affiliations:** Shriners Children’s - Boston; Shriners Children’s - Boston; Shriners Children’s - Boston; Shriners Children’s - Boston; Massachusetts General Hospital; Shriners Children’s - Boston; Shriners Children’s - Boston

## Abstract

**Introduction:**

There has been an emergence of using the antifibrinolytic agent tranexamic acid (TXA) in plastic surgery as an innovative measure to reduce intraoperative bleeding. However, limited data exists regarding topical application of TXA in patients undergoing postburn reconstruction. This study aims to evaluate the effect of topical TXA on blood loss, postoperative complication, and wound healing in postburn reconstructive skin grafting.

**Methods:**

A single-center retrospective cohort study was conducted on all consecutive patients aged 0–21 years old admitted to a pediatric burn center from July 2018 to July 2023 for postburn reconstructive skin grafting by the senior author (D.N.D). The patients in the intervention group received 2.5% TXA applied topically. The primary outcome analyzed was blood loss per cm2 burn area excised. Secondary outcomes included adverse events and postoperative complications such as hematoma, seroma, infection, 2-week delayed wound healing, and 4-week delayed wound healing.

**Results:**

A total of 111 patients were included in this study. For the treatment group, 47 patients received topical TXA during 68 operations on 120 skin graft recipient sites. The control group included 64 patients undergoing 118 operations with 204 skin graft recipient sites. The mean age at reconstruction was 11.7 ± 5.1 years, 53.2% of patients were male, and 44.1% were international referrals. The primary cause of initial injury was flame burn mechanism (73.6%) and the average total body surface area was 43.4 ± 25.0%. Of the 324 skin graft sites, the most common donor site was the thigh, and 63.7% were split-thickness harvested at an average of 14 microns. Patient demographics and reconstructive burn characteristics were similar among both groups (p>0.05).

Skin graft recipient sites that received topical TXA had significantly lower blood loss per cm2 burn area excised (0.27 ± 0.53 ml/cm2 vs 0.66 ± 2.1 ml/cm2, p=0.028). Skin graft recipient sites that received TXA did not exhibit significantly different rates of delayed wound healing at 2-week follow-up (2.5% vs 4.9%, p=0.291) or 4-week follow-up (2.5% vs 2.4%, p=0.982). Rate of hematoma among skin graft recipient sites that received TXA was not significantly different than those that did not (0% vs 0.5%, p=0.442). There were no cases of infection or seroma in either group. No adverse events were observed. Both cohorts had long-term follow-up with an average of 2.5 ± 1.7 years in the control group and 1.1 ± 0.8 years in the TXA group.

**Conclusions:**

Topical TXA may reduce intraoperative bleeding in postburn reconstructive skin grafting with low risk of systemic complications. Of the 120 skin graft recipient sites that received topical TXA, there were no cases of hematoma formation and there was not an increased risk of delayed wound healing.

**Applicability of Research to Practice:**

This is the first study that evaluates topical application of TXA as a promising adjunct in postburn reconstructive skin grafting in children.

**Funding for the Study:**

N/A

